# Green Tea and Red Tea from *Camellia sinensis* Partially Prevented the Motor Deficits and Striatal Oxidative Damage Induced by Hemorrhagic Stroke in Rats

**DOI:** 10.1155/2018/5158724

**Published:** 2018-08-02

**Authors:** Priscila Marques Sosa, Mauren Assis de Souza, Pâmela B. Mello-Carpes

**Affiliations:** ^1^Physiology Research Group, Stress, Memory and Behavior Lab, Federal University of Pampa, Uruguaiana, RS, Brazil; ^2^Multicentric Graduate Program in Physiological Sciences, Federal University of Pampa, Uruguaiana, RS, Brazil; ^3^Biological Sciences: Physiology Graduate Program, Federal University of Rio Grande do Sul, Porto Alegre, RS, Brazil

## Abstract

Green tea from *Camellia sinensis* plays a well-established neuroprotective role in several neurodegenerative diseases, including intracerebral hemorrhage (ICH). However, the other teas of the same plant do not have their properties well understood; but they can be as effective as green tea as an alternative therapy. In this study, we investigated the effects of supplementation with green tea and red tea from *Camellia sinensis* on motor deficits and striatum oxidative damage in rats submitted to hemorrhagic stroke (ICH). Male Wistar rats were supplemented with green tea, red tea, or vehicle for 10 days prior to ICH induction. After injury, the rats were submitted to motor tests (open field for locomotion, rotarod for balance, and neurological deficit scale (NDS)) 1, 3, and 7 days after ICH induction, while the tea supplementation was maintained. Subsequently, the rats were euthanized to striatal tissue dissection for biochemical analyzes (lipid peroxidation, reactive oxygen species, glutathione levels, and total antioxidant capacity). ICH caused locomotor and balance deficits, as well as increased the neurological deficit (NDS). Only red tea prevented locomotor deficits after injury. Green tea and red tea prevented balance deficits on the seventh day after ICH. On NDS evaluation, green tea presented a better neuroprotection than red tea (until day 3 after ICH injury). In addition, ICH increased reactive oxygen species and lipid peroxidation levels, without altering antioxidant markers. Green and red teas were effective in decreasing the lipid peroxidation levels. Therefore, green and red teas partially prevented the motor deficits and striatal oxidative damage induced by ICH. Based on our results, we can consider that the two teas seem to be equally effective to prevent motor deficits and striatal oxidative damage induced by hemorrhagic stroke in rats.

## 1. Introduction

Intracerebral hemorrhage (ICH) is a common type of stroke associated with a considerable socioeconomic impact, disability, and mortality [1] and represents 15 to 20% of all stroke cases [2].

Some regions of the brain are more susceptible to stroke damage, including striatum, which is one of the most important regions for voluntary motor control [3]. After ICH, the hematoma components initiate an inflammatory signaling through the activation of microglia, which culminates on secondary damage [4, 5]. Progressive deterioration of brain tissues is classified as ICH secondary damage and plays an important role in neurological impairment [4]. These molecular events that occur during ICH increase the production of hydroxyl radicals and oxidation of lipids [6], which expose the brain to higher levels of reactive oxygen species (ROS). There is increasing evidence that oxidative stress contributes to ICH-induced secondary brain injury through the generation of ROS [7]. The biochemical events involved in secondary damage are not well described but represent an important therapeutic target after ICH [7].

Despite the breakthrough in research, ICH treatments deserve great attention and further investigation. So, to seek adjuvant therapies that may prevent the progression of ICH secondary damage is important [8]. In this sense, interventions with potential antioxidants are being studied for treatment of damage caused by redox imbalance in brain tissues [9]. Antioxidants from natural products can slow or reverse the damage caused by excessive ROS production, as demonstrated in other models of neurodegenerative diseases related to oxidative damage [10, 11]. Several studies have shown the efficacy of the treatment with teas from *Camellia sinensis* as an antioxidant strategy [11, 12].

Green tea derived from *Camellia sinensis* contains a high content of flavonoids: epigallocatechin-3-gallate (EGCG), which accounts for approximately 59% of its total catechin content, epigallocatechin, epicatechin, and others [13]. Several studies have shown the antioxidant effects attributed to EGCG [14]. However, there are important variations in the herb's processing, and it can lead to changes in the content of flavonoids [15]. So, the different concentrations of catechins in the various types of tea derived from *Camellia sinensis* may be responsible for different neuroprotective effects of each one of them.

Recent studies comparing the therapeutic potential of the different teas of *Camellia sinensis* to protect from memory deficits in ischemia reperfusion (IR) [11] and in an Alzheimer disease model [16] have shown that, in addition to green tea, red tea also exerts an effective neuroprotection in these models. Other study performed by our team has already demonstrated the neuroprotective effect of green tea in ICH models [12]; however, comparative effects between the two teas were not analyzed in this model. Considering that both teas are very popular and accessible, studies comparing the neuroprotective effects of them are important. Here, we determine the neuroprotective potential of green tea and red tea from *Camellia sinensis* in the prevention of possible motor deficits and striatal oxidative damage in a model of intracerebral hemorrhage in adult rats.

## 2. Material and Methods

### 2.1. Animals and Experimental Design

Forty male Wistar rats (250–350 g) were obtained from the Central Vivarium of the Federal University of Santa Maria (UFSM-Santa Maria, RS, Brazil). The rats were housed 4 per cage and kept in a temperature-controlled room (24°C ± 1), with light/dark cycle of 12 h and food and water *ad libitum*. All the experiments were performed in accordance with the standards of the “Principles of laboratory animal care” (NIH publication No. 85-23, revised 1996) and were previously approved by the Institutional Animal Care and Use Committee (protocol #002/2017).

**Figure 1:**
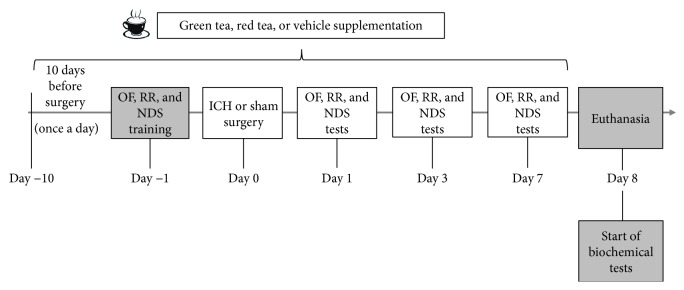
Experimental design. All rats were supplemented with green tea (GT), red tea (RT), or vehicle for 10 days prior to intracerebral hemorrhage (ICH). Twenty-four hours after, the rats were submitted to training in neuromotor tests (open field: OF; rotarod: RR; neurological deficit scale: NDS). In the following day (day 0), the rats were submitted to ICH or sham surgery following by 24 hours of recovery. On days 1, 3, and 7 after surgery, the rats were submitted to neuromotor tests. During all behavioral testing days, the rats continued to receive supplementation with tea or vehicle. On the eighth day, the rats were euthanized and the striatum were isolated to biochemical testing.

The rats were randomly divided into four groups: sham; intracerebral hemorrhage (ICH); intracerebral hemorrhage + green tea (ICH + GT); and intracerebral hemorrhage + red tea (ICH + RT). Rats received specific tea or vehicle for ten days prior to ICH surgery [12]. After, the animals were submitted to the training in neuromotor tests (open field, rotarod, and neuromotor deficit scale) and, in the following day, to the ICH or sham surgery. On days 1, 3, and 7 after surgery, the animals were tested in the neuromotor tests and after were euthanized to striatum dissection and preparation for biochemical essays. During the behavioral test period, the animals continued to receive tea or vehicle supplementation (Figure 1).

### 2.2. ICH Surgery

Surgical procedures were performed aseptically. The rats were anesthetized with ketamine and xylazine (i.p., 75 and 10 mg kg, resp.). Body temperature was maintained at 37°C during surgery using a surgical warming table. After placing the animals on the stereotactic table, a 3.5 mm hole was made to the right and 0.5 mm anterior to bregma. A 26-gauge needle (Hamilton, Hamilton, NV, USA syringe) was inserted unilaterally into 6.5 mm in the right striatum to infuse 1 *μ*l of sterile saline containing 0.2 U of bacterial collagenase (type IV). The needle was left in place for 5 min and then slowly withdrawn. The hole was sealed with a metal screw, suture threads were used to close the wound, and lidocaine was used at the suture site [17]. The same procedure was performed with the sham animals, except that the infusion did not contain collagenase, only saline.

### 2.3. Tea Supplementation

The two types of *Camellia sinensis* teas (green and red) were purchased directly from the same company (Madrugada Alimentos Ltda., Venâncio Aires, RS, Brazil), and prepared daily in the same way.

The teas were prepared with distilled water (95°C ± 5°C) and then the infusion was maintained rested and muffled for approximately 3 minutes, and after was filtered. Teas were administered via gavage (400 mg/ml/day), at room temperature [9].

Tea samples used in this study were analyzed by high-performance liquid chromatography (HPLC) to analyze the presence of epicatechin (EC), epigallocatechin (EGC), epigalocatechin gallate (EGCG), and epicatechin gallate (ECG) [18] (Table 1).

### 2.4. Behavioral Testing

To verify possible changes and/or deficits in motor function, the rats were trained in OF, RR, and NDS tasks 24 h prior to ICH or sham surgery. The tests were performed 24 h, 3 days and 7 days after the surgeries.

### 2.5. Open Field Test (OF)

To evaluate the locomotor activity and the exploratory behavior of the rats, we used the open field apparatus. The open field consists in a box (60 cm in diameter × 45 cm in height) divided into 12 quadrants of the same surface area. The animals were gently placed in the open field arena (i.e., in the box), so that they could freely explore it for 5 min. The number of crossed lines (crossings) was counted [19]. After each rat testing, the arena was cleaned with 70% ethanol.

### 2.6. Rotarod Test (RR)

In order to evaluate the influence of ICH in rats' motor coordination and balance, the RR test was used. The apparatus consists of a rotating cylinder (5 cm diameter × 8 cm wide × 20 cm high), with an automatic fall register. On the training day, the animals were placed in the static cylinder for 2 minutes for habituation. After the habituation time, the cylinder rotation was set at 16 rpm for 6 minutes, and the number of falls and latency for the first fall were recorded. In the test sessions, the animals were suspended for 6 minutes in the cylinder at a speed set in 20 rpm. The number of falls and latency for first fall were again recorded [20].

### 2.7. Neurological Deficit Scale (NDS)

This test set is sensitive to striatal/motor damage [21]. The rats were trained in NDS before surgery, for reference. On test days after surgery, they were resubmitted to NDS. Briefly, the rats were evaluated for spontaneous movement, hind limb retraction, bilateral forefoot grip, contralateral forearm flexion, and beam trajectory. A maximum score of 14 indicates greater neurological impairment [22].

### 2.8. Biochemical Analyses

#### 2.8.1. Tissue Preparation

Rats were sacrificed 24 hours after the last test day. Their brains were removed, and the striatum ipsilateral to the lesion was quickly dissected and homogenized in 50 mM Tris HCl, pH 7.4 (1/10, *w*/*v*). The samples were centrifuged at 2400*g* for 10 min, and the supernatants (S1) were used for assay.

#### 2.8.2. Lipid Peroxidation

Lipid peroxidation was assessed by the substances reactive to thiobarbituric acid (TBARS) test [23]. S1 aliquot was incubated with a solution of 0.8% thiobarbituric acid, acetic acid buffer (pH 3.2), and sodium dodecyl sulfate solution (8%) at 95°C for 2 h. The color reaction was measured at 532 nm. Results were expressed as nmol of malondialdehyde (MDA) per mg protein.

#### 2.8.3. Reactive Oxygen Species (ROS) Levels

ROS levels were determined indirectly by fluorimetric method spectrum using 2′, 7′-dichlorofluorescein diacetate (DCFH-DA). The samples were incubated in the dark with 5 *μ*l of DCFH-DA (1 mM). Oxidation monitoring was made of DCFH-DA to dichlorofluorescein (DCF) fluorescence by reactive oxygen species. The fluorescence emission intensity was performed at 520 nm (with excitation at 480 nm) for 60 minutes after the addition of DCFH-DA in spectrofluorimeter (Shimadzu RF-5301PC Model).

#### 2.8.4. Glutathione (GSH) Levels

GSH levels were fluorometrically determined (Hissin and Hilf, 1976). An aliquot of homogenized sample was mixed (1 : 1) with perchloric acid (HClO_4_) and centrifuged at 3000*g* for 10 min. This mixture was centrifuged, the protein pellet was discarded, and free thiols (SH) groups were determined in the clear supernatant. An aliquot of supernatant was incubated with orthophthaladehyde and fluorescence was measured at excitation of 350 nm and emission of 420 nm. Results were normalized by mg of protein and expressed as percent of control.

#### 2.8.5. Total Antioxidant Capacity

Total antioxidant capacity was measure by ferric reducing/antioxidant power assay (FRAP). Working FRAP reagent was prepared by mixing 25 ml acetate buffer, 2.5 ml TPTZ solution, and 2.5 ml FeCl3.6H2O solution, and 10 *μ*l of homogenate was added in the 300 *μ*l working FRAP reagent in a microplate (Benzie and Strain, 1996). A standard curve with 10 *μ*l trolox (concentrations of 15, 30, 60, 120, and 240 mM) and 300 *μ*l working FRAP reagent was used. The microplate was incubated at 37°C for 15 min before reading in SpectraMax M5 Microplate Reader at 593 nm. Each sample was analyzed in triplicate.

#### 2.8.6. Protein Determination

Protein content was measured colorimetrically by the method of Bradford [24] using bovine serum albumin (1 mg·ml^−1^) as standard.

### 2.9. Statistical Analysis

Data were checked for normality of distribution using Shapiro-Wilk test. Results are presented as mean ± standard error of the mean (SEM). Results were analyzed by one-way ANOVA followed by Tukey's post hoc test, when appropriate. Significance level was set at 0.05 for all analyses.

## 3. Results

### 3.1. Behavioral Assessment

**Figure 2:**
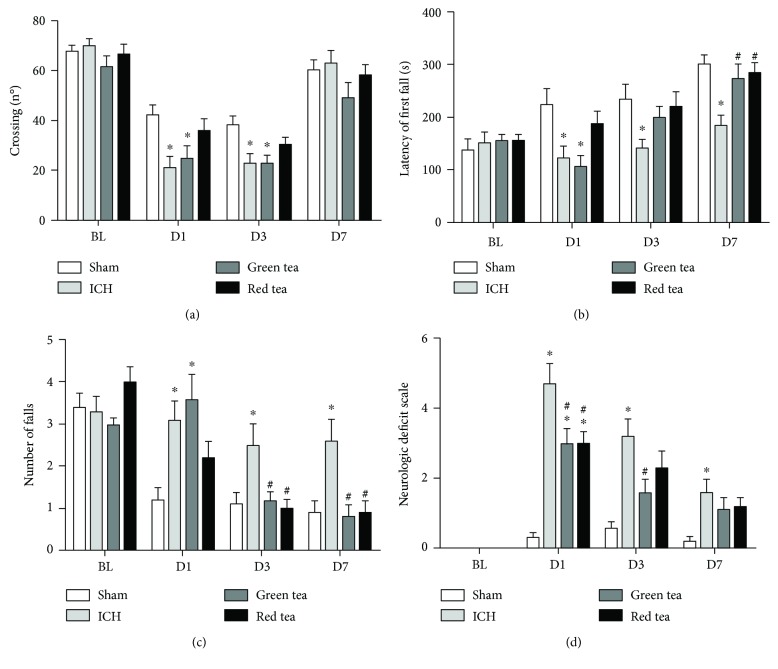
Effects of green and red tea administration on neuromotor function after ICH in rats. (a) Open field test: number of crossings; (b) rotarod test: latency to the first fall in seconds; (c) rotarod test: total number of falls; (d) neurological deficit scale: total score. Data are presented as the mean ± S.E.M. One-way ANOVA ^∗^*P* < 0.05 in comparison to sham group, ^#^*P* < 0.05 in comparison to the ICH group (*n* = 10/group).

**Figure 3:**
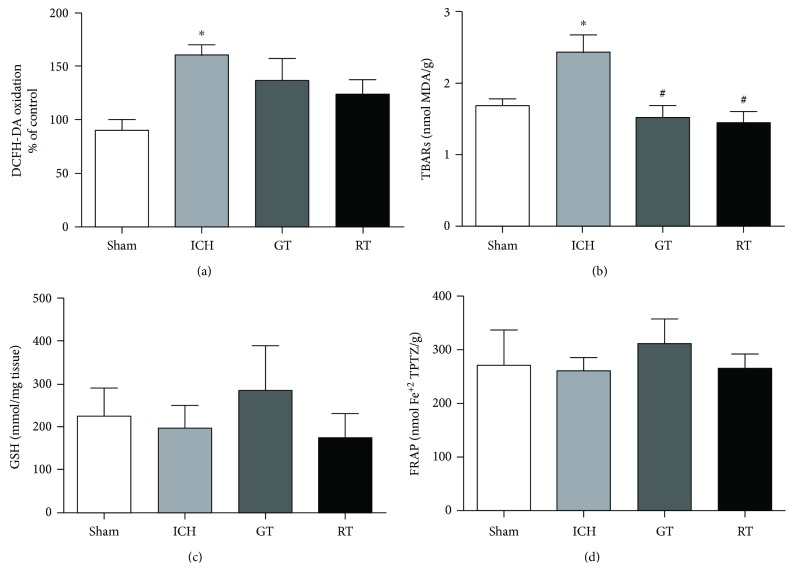
Effects of administration of green tea and red tea on oxidative stress, oxidative damage, and antioxidant markers on striatum after ICH in rats. (a) ROS levels by DCFH method; (b) lipid peroxidation by TBARS (thiobarbituric acid reactive substance); (c) glutathione levels (GSH); (d) total antioxidant capacity by FRAP method. Data are presented as the mean ± S.E.M. One-way ANOVA ^∗^*P* < 0.05 in comparison to sham group; ^#^*P* < 0.05 in comparison to ICH group (*n* = 6/group).

The locomotor activity was assessed using the OF test. The locomotor activity was different between the groups on days 1 (*F*_(3, 36)_ = 4.621, *P* = 0.0078, Figure 2(a)) and 3 (*F*_(3, 36)_ = 4.771, *P* = 0.0067, Figure 2(a)). However, on day 7 there was no difference between the groups (*F*_(3, 36)_ = 1.563, *P* = 0.215, Figure 2(a)). The post hoc test showed that the rats in the ICH group had decreased locomotor activity compared to sham rats on day 1 (*P* < 0.05, Figure 2(a)) and day 3 (*P* < 0.05, Figure 2(a)). On day 7, there was no difference in locomotor activity between the ICH and sham rats (*P* > 0.05). In addition, there are no differences on crossings between ICH and ICH + green tea groups on days 1 and 3 (*P* > 0.05, ICH versus ICH + teas Figure 2(a)). Importantly, ICH + red tea presented similar number of crossing to the control group on both days (D1: *P* = 0.0078; D3: *P* = 0.0067), while green tea did not present the same effect.

In rotarod test, there was a difference between the groups on days 1 (*F*_(3, 36)_ = 5.264, *P* = 0.004, Figure 2(b)), 3 (*F*_(3, 36)_ = 2.925, *P* = 0.046, Figure 2(b)), and 7 (*F*_(3, 36)_ = 6.352, *P* = 0.001, Figure 2(b)), considering the latency for the first fall. The rats in the ICH group presented a deficit in the balance (shorter latency to the first fall than control animals) on days 1, 3, and 7 (*P* < 0.05, Figure 2(b)). GT and RT supplementation showed a neuroprotective effect on the seventh day (*P* < 0.05 for GT; *P* < 0.01 for RT; Figure 2(b)). In addition, there were no differences in the balance between ICH + ICH tea groups on days 1 and 3 (*P* > 0.05, ICH versus ICH + teas; Figure 2(b)), but ICH + RT presented a similar latency for the first fall than the control group on day 1 (*P* = 0.04, Figure 2(b)).

Considering the number of falls (rotarod test), we observed difference between the groups on days 1 (*F*_(3, 36)_ = 5.698, *P* = 0.0027, Figure 2(c)), 3 (*F*_(3, 36)_ = 4.832, *P* = 0.0063, Figure 2(c)), and 7 (*F*_(3, 36)_ = 5.921, *P* = 0.002, Figure 2(c)). The ICH rats showed a higher number of falls than sham rats on days 1, 3, and 7 (*P* < 0.05, Figure 2(c)). The GT reversed this deficit at days 3 (*P* < 0.05, Figure 2(c)) and 7 (*P* < 0.01, Figure 2(c)), as well as RT (D3: *P* < 0.05; D7: *P* < 0.01; Figure 2(c)), but only ICH + red tea presented similar number of falls to the control group on day 1 (*P* > 0.05, Figure 2(c)).

On neurological deficit (NDS) evaluation, we found differences between the groups on days 1 (*F*_(3.36)_ = 20.47, *P* < 0.0001, Figure 2(d)), 3 (*F*_(3.36)_ = 7.704, *P* = 0.0004, Figure 2(d)), and 7 (*F*_(3.36)_ = 4.21, *P* = 0.013, Figure 2(d)). ICH rats presented higher NDS score than sham rats on days 1 (*P* < 0.0001, Figure 2(d)), 3 (*P* < 0.001, Figure 2(d)), and 7 (*P* < 0.05, Figure 2(d)). The GT and RT were able to reverse these deficits on day 1 (GT: *P* < 0.05, RT: *P* < 0.05, Figure 2(d)), but, on day 3, only GT reversed the neurological deficit (*P* < 0.05, Figure 2(d)).

### 3.2. Biochemical Assays

Considering that the increase of oxidative stress is one of the secondary damage characteristics of ICH affected tissues, we evaluated the levels of oxidative species, the oxidative damage, the levels of GSH (an antioxidant marker), and the total antioxidant capacity in the striatum of rats.

A significant difference on the striatal level of oxidative species (DCFH) (*F*_(3, 20)_ = 6.850, *P* = 0.0137, Figure 3(a)) was observed. The post hoc showed that ICH rats presented increased ROS compared to the sham group (*P* < 0.01). Neither green tea (*P* > 0.05) nor red tea (*P* > 0.05) was able to reverse striatal oxidative stress.

There was difference between the groups on lipoperoxidation (TBARS) (*F*_(3, 20)_ = 7.144; *P* = 0.0019, Figure 3(b)). ICH rats showed an increase in lipoperoxidation compared to sham rats (*P* < 0.05). Both green tea and red tea avoided this increase (GT: *P* < 0.01 versus ICH; RT: *P* < 0.01*versus* ICH; Figure 3(b)).

Considering the antioxidant measures, we did not find differences between the groups in the GSH levels (*F*_(3, 20)_ = 0.4608, *P* = 0.7127, Figure 3(c)), as in the total antioxidant capacity (FRAP; *F*_(3, 20)_ = 0.2767, *P* = 0.8415, Figure 3(d)).

## 4. Discussion

In this study, we determined the neuroprotective potential of green tea and red tea on the motor deficits and striatal damage caused by hemorrhagic stroke in rats. Our major findings demonstrate that the two teas studied partially protect against neuromotor deficits and oxidative damage caused by ICH, and appear to be promoters of neuroprotection in this model. The different teas protect against neurological deficits mainly on the first and third day after the injury, avoiding locomotor (OF) and balance deficits (RR), general neuromotor function deficits (NDS), and contributing to avoid the increase of lipid peroxidation (TBARS).

ICH is associated with motor damage [25, 26] and cognitive impairments [27] that may be closely related to oxidative stress [28]. Our current results show that ICH generates locomotor and exploratory damage (OF), which is maintained for up to 3 days after injury. However, in this case, only the red tea was able to protect against such damage. A significant finding is that on the seventh day after the injury, a spontaneous recovery was observed in relation to the locomotor and exploratory activity, which is in agreement with Lu et al. [29], but prevents us from evaluating whether the teas have a protective effect in this case. Additionally, the results show that ICH rats presented a balance impairment (RR) until the seventh day after ICH, and that the green and red teas were able to protect against such damages on days 3 and 7 after the injury. The neuromotor deficit was also observed from the first to the seventh day after ICH in NDS test [22]. Green tea was effective to protect the damage on test day 1 and 3 after injury, while red tea was effective only on test day 1.

Strategies that propose the administration of antioxidant agents have been widely tested to prevent or treat hemorrhagic stroke [30, 31]. Previous studies have already demonstrated the neuroprotective effects of green tea on balance in ICH [12] and brain ischemia-reperfusion models [18, 32]. It is known that ICH injury mechanisms are related to increased free radical production and consequent oxidative stress [9, 28]. In this study, ICH induced an increase on striatum ROS and on lipid peroxidation. Green tea and red tea were able to protect the striatum tissue against lipid peroxidation. Any alteration on antioxidant markers was detected.

The administration of tea from *Camellia sinensis* and EGCG (the main component of green tea) has already been described as neuroprotective by its antioxidant effects in different models of brain injury [9, 28]. A recent study conducted in our laboratory that sought to evaluate the neuroprotective capacity of 4 teas from *Camellia sinensis* (green, red, white, and black) in an ischemic stroke model showed that the greatest protective effect on memory and oxidative stress is from green tea and red tea, since the best effects associated with green tea [11]. In another study using green, red, and black teas from *Camellia sinensis*, Schimidt and colleagues suggest that supplementation with green tea and red tea may avoid deficits in social and object recognition memories related to Alzheimer's disease, but only green tea avoids the hippocampal oxidative stress and damage induced in Alzheimer model [16]. These effects may be closely related to the greater amount of specific catechins present in green tea [33, 34], since some literature data suggest that the beneficial effects of teas are mainly related to catechins polyphenol and their derivatives [35]. However, this may not be the only explanation for their ability to neuroprotection. In addition to the antioxidant capacity, we cannot disregard the anti-inflammatory potential and inhibition of acetylcholinesterase action [36] of the *Camellia sinensis* teas, although these mechanisms are not completely clear.

The great amount of compounds in teas emphasizes the differences of mixture use or its isolated components. Here, considering that the neuroprotection effects could be related to the combined activity of the varios teas' compounds [37], we decided investigated the effects of the teas, and not of the isolated compounds, mainly because the teas mixture are available to population.

## 5. Conclusions

Green and red teas have been shown to be effective strategies, with similar effectiveness, to prevent some motor deficits and the striatal oxidative damage induced by ICH. The mechanisms involved in their protective role include the decrease of oxidative damage, that is, lipid peroxidation.

## Figures and Tables

**Table 1:** Concentration of catechins (mg/ml) found in samples of the two teas from *Camellia sinensis.*

Catechins	Green tea	Red tea
(−)-Epigallocatechin	6412.14	ND
(−)-Epicatechin	5736.00	5442.34
(−)-Epigallocatechin gallate	9405.42	ND
(−)-Epicatechin gallate	2609.19	ND

ND: not detected.

## Data Availability

The data can be made available through request by e-mail pamelacarpes@unipampa.edu.br.

## Additional Points

*Highlights*. Hemorrhagic stroke (ICH) causes motor deficits and oxidative damage in the striatum. Green tea and red tea partially avoided the motor deficits caused by ICH. Green tea and red tea avoided the striatal oxidative damage caused by ICH.
